# Soft Foil and Progressive Dentistry

**Published:** 1889-05

**Authors:** E. B. Davis

**Affiliations:** Concord, N.H.


### SOFT FOIL AND PROGRESSIVE DENTISTRY.[1]

BY E. B. DAVIS, D.D.S., CONCORD, N. H.

The last few years have seen the most important improvements in the science and art of dentistry, and the deepest and most conclusive investigations into the histological and pathological branches of the study that have ever taken place. In fact, I think no other professsion will show an equal progress in its deeper branches in the same length of time.

Even from my own standpoint of only fifteen years the progress is almost incredible, and it is only by closely observing the steps that the profession has taken in its advance, that we are able to keep up with the procession in its onward march towards the high mark, set by the men eminent in ability, and who would, from their varied accomplishments and talents, adorn any position in life. The leaders in American dentistry have been leaders in every sense of the word.

To-day is a day of progress. The times are pregnant with that " one idea " progress. No man at the present time can do himself,

---

[1] Read before the New England Dental Society, November 15, 1888.

or the cause which he champions any credit, but through the most earnest efforts to keep up with the advance that is taking place in this, as well as other professions.

That very stormy discussion on the so-called "new departure in filling teeth," commenced about ten years ago, by Professor Flagg, I think, taught us a great deal in regard to the use of plastics, and, as far as I know, although no number of men accepted the extreme views advocated, yet a great many profited by the discussion, and now use better plastic fillings, and use them more intelligently.

The long and very able discussion on pyorrhœa alveolaris has aroused the profession into taking a deeper interest in that very aggravating disease, and our progress in treating it is a long way in advance of the position occupied a few years ago.

The very able discussion and papers on crown and bridge-work that have occupied our attention as a profesion for several years, will ultimately land us in a position far in advance of that which we occupied before they were commenced.

The very important investigations that have been taking place in regard to the etiology of decay have, at last, we have reason to believe, come upon solid ground, and from the progress of the investigations results will be obtained by the profession for which it has so long tried, namely: something to prevent decay in teeth, in a measure at least; yet not to the extent that all dentists will go out of business, but that much suffering will be prevented, and many of the abominations in the form of prosthetic dentistry will no longer disgrace the features of the American people, and that will, in the highest sense, be a step in the direction of progress such as the whole civilized people will delight to see.

Each and all of the men engaged in these investigations, and others of less practical importance, are doing a grand work for humanity, which will be felt for all time to come; and we cannot do less than give them the credit due, and honor them for the work they have done, and are still doing. I think, and all will concede, that dentistry is a very progressive profession, and takes but few, if any, steps backward.

I have been led into taking the subject that I have chosen for this paper, "Soft Foil and Progressive Dentistry," by several considerations. And first is the fact that we have all heard the distant murmurings of a recurrence of this, one of the oldest and most hotly contested of dental discussions. It has been coming nearer, and I think has finally been placed before the profession by one of the leaders in this very branch of our work, Prof. James Truman, of Philadelphia, in a paper read before the Pennsylvania State Dental Society. And another consideration was, that I have never found a young dental operator, that claimed to be an expert, or even understood the first principles of working soft foil, although not one would admit that he was not expert in cohesive foil working, and took great delight in criticizing the work of others in this branch of our profession.

Another thing that brought this subject to my mind was the very severe criticism by a dentist of Washington, D.C., in the Dental Office and Laboratory for July of this year. It was this: "I believe cohesive foil in the hands of two-thirds of the operators of the present day is a delusion and a snare." And I thought if this is what the dental profession has come to, with all its boast of progress, after over thirty years of work on this one branch of our profession, the very branch that has received the most practical attention of any in the whole range of our practice, surely this severe and sweeping criticism should not be accepted without the closest examination.

I confess I cannot affirm or deny this statement, and will leave it for those that come after me; but I will state right here that I am, and always have been, a thorough believer in soft foil, and from my own standpoint it does not strike home very forcibly, though there is no one who regrets it more than I, if it is true, or even has any foundation in fact.

The murmurings that I have spoken of come to us in this form: It is conceded by able men in the profession that soft foil is good to line cavities with. Use just a little of it, and make the bulk of the filling of cohesive foil. Why not use the larger amount of soft and the lesser of cohesive foil? And that is what the next step will be. The profession is having its attention called to this form of filling by leading operators, and when the profession generally comes to understand the easy and rapid work that can be done by an expert soft-gold operator, in comparison with the slow, and oft-times tedious method of the cohesive gold operator, I think a gradual change will take place in the mode of using gold as a filling material.

I don't think there can be any question in regard to soft gold not being a good tooth-saving material. I thing that the expert operators of thirty or more years ago have demonstrated to all succeeding generations that it comes the nearest to an ideal filling material for a large number of cases of any that we have ever had. It can be inserted rapidly, condensed solidly, without the use, in many cases of the rubber dam, and will prevent the recurrent decay in a manner that cohesive gold, from defective manipulation, oftentimes fails to do. And, furthermore, I think that the large number of dentists that learned to use soft gold in a thorough manner, and have never discontinued its use, but have used the other forms of gold in connection with it, have been doing better by their patients than a majority of those who have been doing an exclusive cohesive foil practice. And I am sure these men will hail with delight the evidence that some of the leading operators are again receiving this old and dear friend to their practice, acknowledging its merits, and using it in those cases where its use has proved of so much value in preventing recurrent decay. I look upon this return to the use of soft gold as an act of progress in our profession.

It is not my object or purpose in this paper to disparage cohesive foil as a filling material. There are many cases where it is preferable and where no other form of gold should be used. And I say that it should be used in cases where it ought to be used, and soft foil in all cases where it can be used. I have filled but very few cavities without using cohesive gold in connection with the soft gold, but the amount of soft foil used has greatly predominated over the cohesive. By this method contour can be restored as well, and in much less time than by the old cohesive method. Grinding surfaces, and those surfaces to be exposed and highly finished, are in all cases to be of cohesive foil; but as a young man, I wish to place myself on record as a strong champion of soft foil, alongside of many of the old practitioners of the day.

In any profession or trade it is right for a man, when he finds a material that he can use successfully, and knows what he can do with it under all circumstances, so long as the standard of excellence is maintained, to stick to that material in preference to continually experimenting with all the different materials that may be brought to his attention; and it is not my object to try to influence any man to change his mode of practice, if he is satisfied with the results he is attaining with cohesive gold, and can afford to spend from thirty minutes to one hour in filling a cavity that could by the use of soft gold by an expert operator be filled in ten or fifteen minutes. For, in the discussions and papers that have of late been given on this subject, time is one of the principal reasons given in favor of returning to the use of soft gold.

And, furthermore, I would like to inquire if the very general use of cohesive foil for the last twenty-five years has so fully demonstrated to us that it is the best form of foil to use in all cases, and by all operators; that there is no need of any other form but cohesive, and if their belief in cohesive gold is growing stronger as time and experience go on?

If I thought these questions could be answered in the affirmative, I could have no object in presenting this paper to you to-day. I think the facts and evidence are not wholly in favor of cohesive gold for filling teeth.

We cannot expect the profession to "about face" on this subject, and after the long and honorable stand it has occupied in favor of cohesive gold, the return to the use of soft gold will be gradual, and the more certain for that. The first steps have been taken by leading men in the profession advocating the use of soft gold in lining cavities, the larger part of the filling being built up of cohesive gold. This is a very important step, and one that recognizes the peculiar virtues of soft foil, and, to my mind, the beginning of a more general use of this form of gold as its working qualities become better known to this generation of dentists.

This advice in regard to the use of soft gold has been thrown out in a very gentle manner, and not in a way to attract the attention of the profession generally. At the dental conventions that I have attended of late this subject has received little or no attention, either by essayists or in discussion; but at the meeting of American Dental Association, held in this city in 1880, I listened to a very interesting discussion on it. The advocates of cohesive gold were led by the lamented Dr. M. H. Webb, full of faith that that was the only correct method of using gold, and that all failures from it were the result of defective manipulation. Opposed to him were the older members of the profession, still in love with the material that they had learned to use when it required years of time to become expert with it, owing to the want of appliances that we have at the present day.

The discussion was intensely interesting to me, and from the knowledge I possessed of both methods, I could appreciate the points brought out, and notwithstanding the fact that the cohesive gold side was most ably presented, I could not be persuaded to think that cohesive foil was the only form in which gold should be used to prevent recurrent decay in teeth; and the longer the discussion continued, the stronger I became and the more faith I had in the teachings I had received as a student, to wit: that in a large majority of cavities where gold should be used for the prevention of the further progress of decay, soft foil is the best form of gold to use. The experience of eight years of practice since that time has not changed my opinion in regard to this subject.

In the paper by Prof. Truman that I have referred to, he finally concedes the fact that a good filling can be made with soft and cohesive foil used in connection, and, further, that perhaps it is the best way to fill teeth, all things considered. The only serious objection he raises against soft foil is that it is difficult to teach the principles of using soft gold, while the cohesive foil method can be taught to the man of the dullest comprehension.

The place for the student to acquire the methods and principles of using soft gold is with his preceptor, if he has one, and not leave it for the professor of Operative Dentistry to teach him all his methods of practice.

One statement made in his paper, however, I would question: he says; "Any one who has mastered the art of manipulating cohesive foil cannot fail in the use of soft foil." And further: "The transition from cohesive gold is easy and natural, the former having been properly acquired; but the change from soft to cohesive is almost an impossibility."

It may be presumptive (for me) to question the statements of such a man as Prof. Truman, but all my teachings, and the results of my experience have proved to me the exact reverse. And in proof of this side of the question, I would cite the fact that those operators are able to do equally good work with any form of gold; while, on the other hand, thousands of dentists who do good work in cohesive foil are totally incapable of working in soft gold, and know nothing of its peculiarities. I affirm that an operator who can use soft foil expertly can use any kind of gold in any manner, or any other filling material that we have had thus far in our profession.

In evidence that I have from some of our leading professors of Operative Dentistry in our Dental colleges, I learn that they are teaching the use of soft foil to a considerable extent, some of the professors not considering a student competent to enter upon practice until he is qualified to use gold in all its forms. If a student comes to a college from a preceptor who uses both soft and cohesive gold, and has acquired a knowledge of working gold in all its forms, his advance will be as easy and rapid as his qualifications will admit of; but, on the other hand, if he has acquired during his studentship a knowledge of cohesive gold only, the college should do all in its power to perfect him in soft gold manipulation.

